# A Novel Ultrasound Technique Based on Piezoelectric Diaphragms Applied to Material Removal Monitoring in the Grinding Process

**DOI:** 10.3390/s19183932

**Published:** 2019-09-12

**Authors:** Felipe A. Alexandre, Paulo R. Aguiar, Reinaldo Götz, Martin Antonio Aulestia Viera, Thiago Glissoi Lopes, Eduardo Carlos Bianchi

**Affiliations:** 1 Department of Electrical Engineering, São Paulo State University—UNESP, Av. Eng. Luiz Edmundo Carrijo Coube, 14-01, Bauru 17033-360, Brazil; paulo.aguiar@unesp.br (P.R.A.); reinaldo.gotz@usp.br (R.G.); martin.aulestia@unesp.br (M.A.A.V.); thiago.glissoi@unesp.br (T.G.L.); 2 Department of Mechanical Engineering, São Paulo State University—UNESP, Av. Eng. Luiz Edmundo Carrijo Coube, 14-01, Bauru 17033-360, Brazil; bianchi@feb.unesp.br

**Keywords:** ultrasound technique, grinding monitoring, digital signal processing, piezoelectric transducers

## Abstract

The interest of the scientific community for ultrasound techniques has increased in recent years due to its wide range of applications. A continuous effort of researchers and industries has been made in order to improve and increase the applicability of non-destructive evaluations (NDE). In this context, the monitoring of manufacturing processes, such as the grinding process, arises. This work proposes a novel technique of ultrasound monitoring (chirp-through-transmission) through low-cost piezoelectric diaphragms and digital signal processing. The proposed technique was applied to the monitoring of material removal during the grinding process. The technique is based on changes in ultrasonic waves when propagated through the material under study, with the difference that this technique does not use traditional parameters of ultrasonic techniques but digital signal processing (RMS and Counts). Furthermore, the novelty of the proposed technique is also the use of low-cost piezoelectric diaphragms in the emission and reception of ultrasonic waves, enabling the implementation of a low-cost monitoring system. The results show that the monitoring technique proposed in this work, when used in conjunction with the frequency band selection, is sensitive to the material removal in the grinding process and therefore presents an advance for monitoring the grinding processes.

## 1. Introduction

The manufacturing processes convert raw materials into finished products, often employing machines or machine tools [1]. Among the finishing processes, grinding is considered one of the most important processes because it is applied in the final stage of the production line, providing the final finishing of the part [2].

In the grinding process, the material removal mechanism is performed by the contact between the abrasive surface of the grinding wheel, which is composed of thousands of abrasive grains with undefined cutting geometries randomly distributed [3], with the workpiece surface, which makes grinding a highly stochastic process [4]. The monitoring of the grinding process allows a better understanding of the interaction between the grinding wheel abrasive grains and the workpiece surface [5]. In addition, a better understanding of the material removal mechanism can help in the control of the grinding wheel wear and the workpiece surface quality [6].

The monitoring methods used in manufacturing processes are usually separated into two major categories—direct and indirect [7]. Direct methods are usually employed in a laboratory environment because of their intrinsic handling limitations and are less used in industrial environments. On the other hand, indirect methods are more employed in industrial applications because of their easy handling and application, which are very important characteristics in this type of monitoring [8].

In the indirect methods, sensors are employed to measure a certain variable of interest. Among the sensors employed, the most common is the acoustic emission (AE) sensor, used by Nascimento Lopes et al. [9] in the monitoring of the dressing process; the power sensor, used by Chen et al. [10] in the study of the energy efficiency of an industrial cutting process; the vibration sensor (using accelerometers), used by Dimla [11] in the study of the cutting tool wear; and the force sensor (using dynamometers), used by Agarwal et al. [5], along with the acoustic emission sensor, in the monitoring of the grinding process.

The piezoelectric diaphragm (buzzer) is presented as a low-cost alternative to the traditional components used in structural impedance analysis. Freitas et al. [12] studied the feasibility of applying this transducer as an alternative to commonly used transducers in electromechanical impedance (EMI) methods. Budoya et al. [13] employed the piezoelectric diaphragm in the structural health monitoring system (SHM) to detect structural damages through the impedance signatures, acquired during the evaluating process. The piezoelectric diaphragm was also used as an alternative to the traditional vibration sensor (accelerometer), as presented by Lucas et al. [14], in the detection of structural anomalies in induction motors. In addition to this type of monitoring, the piezoelectric diaphragm was used in the monitoring of manufacturing processes, as presented by Marchi et al. [15], in which the electromechanical impedance method was implemented by means of piezoelectric diaphragms in the evaluation of the workpiece surface quality in the grinding process. In Ribeiro et al. [16], the piezoelectric diaphragm was employed to detect the burning phenomenon in ground workpieces, obtaining good results in the use of this transducer as an alternative to the commercial AE sensor.

This work proposes a new method for monitoring the material removal mechanism in the grinding process by means of the application of piezoelectric diaphragms, employing an alternative through-transmission technique, referred to as chirp-through-transmission ultrasound technique, along with digital signal processing techniques. The present work is an expansion of the research described in Alexandre et al. [17], where initial results were presented. In addition to the use of low-cost piezoelectric diaphragms in the emission and reception of ultrasound waves and the chirp signal as the input to the emitter transducer, what differentiates the proposed technique from others is the digital signal processing of the received signal (RMS and Counts) within a selected frequency band instead of traditional parameters of ultrasound techniques. Thus, this work paves the way to new possibilities for monitoring the material removal mechanism in the grinding process as well as for its automation. It is worth mentioning that the technique presented in this work is a novel approach to the monitoring of the grinding process, as no similar studies were found in literature.

## 2. Traditional Monitoring Techniques Applied to Industrial Processes

The use of sensors in the monitoring of manufacturing processes is consolidated in the manufacturing industry. Some monitoring techniques, such as the passive and active-passive, are well known. In this section, a brief review of researches that made use of such techniques is presented.

### 2.1. Passive Monitoring Techniques

The grinding process is a complex manufacturing process influenced by many factors, such as the workpiece, the grinding machine, the grinding wheel and the process parameters [15]. Due to the complexity of the process, grinding monitoring is very important for its automation and optimization. The use of different sensors in the passive configuration, such as the acoustic emission sensor and the piezoelectric diaphragm along with digital signal processing techniques, allows the detection of events in the grinding process and other industrial processes. Some advantages of the piezoelectric diaphragm can be herein highlighted as follow. The cost of the sensor is one of the main factors that makes the piezoelectric diaphragm more attractive than the acoustic emission sensor. This transducer has a very simple construction, consisting of a thin layer of piezoelectric material (active element) adhered to a circular metal plate (diaphragm). The ceramic is coated with a thin metallic film (usually silver) that acts as an electrode [12]. The PZT diaphragms can operate in both active and passive configurations and have an average cost of a few cents versus the high average cost of AE sensors, which range from hundreds to thousands of dollars [18]. In addition, they are compact, flexible, lightweight and simple acoustic components widely used in various electronic devices to produce sound (alarm, ringing and beep) [12,19]. Furthermore, the fastening of the PZT sensor generally requires only a thin layer of cyanoacrylate glue, while for some AE sensors it may be necessary to machine the holder in order to fit the sensor, which is difficult in some cases. In addition, the data acquisition system for the piezoelectric diaphragm has a lower cost compared to the system required for the AE sensor (this is mainly due to the lower frequency response of the PZT sensor). Finally, the PZT diaphragm is highly available as it can be easily bought by users. Although the sensitivity of the low-cost piezoelectric diaphragm is generally lower than the acoustic emission sensors, it can be considered as an alternative for monitoring several applications, as presented in Castro et al. [20], Viera et al. [21] and Ribeiro et al. [16].

The monitoring of the grinding wheel wear has been the subject of several studies since a worn grinding wheel can directly influence on the geometry and quality of the ground surfaces [18]. The most widely used sensors for grinding wheel wear monitoring (macro and micro effects) are acoustic emission, temperature, power and force sensors. The acoustic emission sensor is used for various purposes, such as monitoring the grinding wheel condition during the grinding and dressing process and detecting contact between the grinding wheel and the workpiece [22]. In this context, de Oliveira et al. [23] presented a monitoring system for the grinding process based on the analysis of the acoustic emission signal (RMS) and the construction of acoustic maps. This innovative system enables the evaluation of the dressing process, the topographic mapping of the grinding surface, as well as the interaction between the grinding wheel and the workpiece during the grinding operation.

The approach in which the PZT (buzzer) and the acoustic emission sensor are employed in conjunction with digital signal processing techniques can be found in many studies, where the objective is the monitoring of the grinding process and related processes (dressing). In order to detect the workpiece burning during the grinding process, Ribeiro et al. [16] collected the acoustic activity of the grinding process with a piezoelectric diaphragm and a commercial acoustic emission sensor. The results demonstrate that the selection of frequency bands optimized the use of metrics, such as the RMS and RMSD, in the detection of the burning phenomenon during grinding. In addition, it demonstrates that the piezoelectric diaphragm can achieve similar results to the AE sensor in detecting surface burns on ground workpieces.

A comparative study using a low-cost piezoelectric diaphragm and a commercial acoustic emission sensor, along with digital signal processing techniques in the evaluation of the surface quality of ground ceramic workpieces, was developed by Viera et al. [21,24]. The results showed that the proposed technique (statistic ROP_STFT_) was able to detect roughness variations on the surfaces of the ground workpieces.

Castro et al. [20] employed the piezoelectric diaphragm as an alternative to the acoustic emission sensor in the detection of partial discharges in power transformers. The results showed that the low-cost sensor has low sensitivity when compared to the conventional acoustic emission sensor, however, for the application studied, the collected signals from both sensors had similar behavior, which characterizes its application in the detection of partial discharges in power transformers.

### 2.2. Active Monitoring with Emission-Reception Techniques

The elastic waves, when propagated in a material that presents intrinsic changes, are dispersed in all directions. The active monitoring methods employ fixed transducers at specific locations of the structure to generate and collect elastic waves after propagating through the material [25]. The active monitoring techniques typically employ ultrasonic [25] and piezoelectric transducers [26]. The following sections describe the most important piezoelectric-active techniques applied to processes monitoring.

#### 2.2.1. Electromechanical Impedance Method (EMI) and Frequency Response Function Method (FRF)

The electromechanical impedance method (EMI) has been widely studied for the development of structural health monitoring systems [27]. According to Castro et al. [28], the EMI technique is based on the piezoelectric effect, which allows the monitoring of structural conditions through the electrical impedance of the transducer connected to a structure, since the electrical impedance of the piezoelectric transducer is related to the mechanical properties of the structure. The EMI technique can be applied to different types of materials, such as metallic materials and composites. For example, highlighting the works of Na et al. [29] on composite structure, Annamdas et al. [30] on concrete structure and Zhu et al. [31] on steel structure, thus indicating the wide range of applications. Structural damage is typically characterized using damage indexes. According to Budoya et al. [32], the most common damage indexes are based on the comparison between two electrical impedance signatures, where one of them is obtained when the structure is considered healthy. The most common indices in SHM analysis are the root mean square deviation (RMSD) and the correlation coefficient deviation metric (CCDM) [33]. The RMSD is indicated to quantify the amplitude differences between two spectrums (baseline and damage) [34], while the CCDM is appropriate to measure the variations or displacement between the frequencies of the analyzed signals [35].

In order to assess the damage present in ground 1020 steel workpieces, Marchi et al. [15] obtained the electromechanical impedance levels for the workpiece without damage and after the grinding process, which made the comparison possible. To assess the damage caused by the grinding process, the results of the RMSD and CCDM indexes were compared with the microhardness and surface roughness of the workpieces.

de Oliveira Conceição Jr. et al. [18,36] assessed the structural changes caused by wear in single-point dressing tools during their lifetime employing EMI method to ensure the reliable monitoring of the tool condition in grinding operations. Representative damage indices, such as RMSD and CCDM, obtained from impedance signatures at different frequency bands were computed for diverse tool conditions. Moreover, an intelligent system, implemented on the basis of artificial neural networks, was able to select the most damage-sensitive features based on the optimal frequency band. The authors highlighted that the EMI method was capable of effectively detect damages in the relatively small diamond tool tips showing a general overall classification error lower than 2% for the best neural models.

In contrast to the conventional EMI method, which is known for using a single transducer, the frequency response function (FRF) method uses at least two transducers, each operating separately as an actuator and as a sensor [37]. Two transducers are employed in this method, one in the emitter mode and the other one in the receiver mode. A significant change caused by incipient damage (detachment, rust, cracks, etc.) results in a difference in the FRF response for an application of the electromechanical impedance technique in an electronic circuit equipped with piezoelectric elements [38]. Liang et al. [39] employed the FRF method in the study of the loosening monitoring of threaded plumbing structures. The use of this method along with the RMSD index allowed the correlation between the RMSD values and the severity of the loosening found in the threaded structures.

#### 2.2.2. Transmitter-Receiver Arrangements for Ultrasonic Inspection

**Figure 1:**
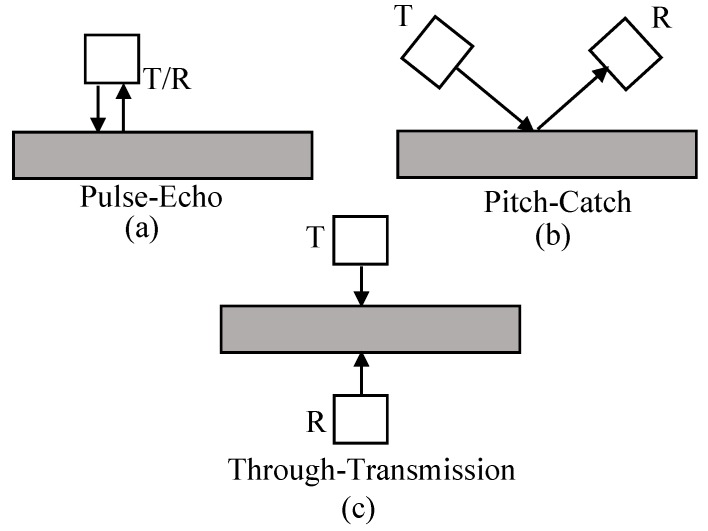
Three most common transmission-reception configurations: (**a**) pulse-echo; (**b**) pitch-catch; and (**c**) through-transmission [41].

The ultrasonic inspection of a component can be performed through two methods—active sensing and passive sensing [40]. In active sensing, a transmitter and a receiver are attached to the structure of interest. In this configuration, the transmitter is responsible for transmitting the signal and the receiver is responsible for receiving the signal. The presence of damage in the region between the transmitter and the receiver causes changes in the ultrasound signal. The damaged region can be identified by means of the analysis of the received signal [41]. The three most common configurations used in ultrasound wave analysis are shown in Figure 1.

Figure 1a,c show configurations based on bulk waves. This type of wave is used when analyzing defects in the internal region of the sample. Figure 1b shows the configuration in which surface waves are used, which detect anomalies on the sample surface. In this work, bulk waves are prevalent due to the transducer configuration, which is similar to the configuration of Figure 1c.

In the pulse-echo system (Figure 1a) the transducer is positioned on the surface of the workpiece. In this method, the same transducer functions as a transmitter and receiver of ultrasound waves [42]. The use of this system for the monitoring of the temperature distribution in the inner region of materials was presented by Ihara et al. [43,44]. The authors applied the proposed technique on heated steel discs at different temperatures. The results prove the feasibility of the technique, the values obtained were consistent with the values measured by thermocouples installed in the sample, thus indicating the effectiveness of the technique.

In the pitch-catch technique, the system has two transducers, the first emits the ultrasound pulses while the second transducer detects the pulses, as shown in Figure 1b. The transducers can be aligned in different ways, such as in normal incidence or oblique incidence, that is, with a certain inclination angle. The technique allows the monitoring of local damages through the emission of known input signals, adequate to diagnose a structure regarding the existence of damage [40]. A study was developed by Ihn et al. [40] employing the pitch-catch technique in the structural health monitoring of an airplane fuselage. The results showed that the use of a damage index allowed the evaluation of damage, similar to the results obtained by the Eddy current method or by the ultrasonic scanning.

The through-transmission technique, similar to the pitch-catch technique, also uses two transducers; however, the positioning of the transducers in the structure is different, as shown in Figure 1c. This technique uses two transducers positioned on opposite sides of the sample. A pulse generator is used to generate continuous electrical pulses with specific frequencies and amplitudes, which are converted into ultrasound waves by the first transducer (transmitter). The ultrasound waves, propagated through the sample, are detected by the second transducer (receiver) [42]. In Raišutis et al. [45], the through-transmission technique was used to estimate the phase velocity dispersion of ultrasound waves in plastic materials. The monitoring of lactic acid fermentation by means of the through-transmission technique was presented by Resa et al. [46]. Through the ultrasonic velocity, it was possible to detect the changes that occurred during the fermentation of carbohydrates. Thus, this non-invasive technique presents great potential in the monitoring of biological processes.

## 3. RMS and Counts in AE Signal Processing

One of the most used statistics in the analysis of the acoustic emission signal is the root mean square (RMS). The application of the RMS statistic in the identification of failures in the grinding and dressing processes has been extensively studied in the specific literature [47,48,49,50]. According to Webster et al. [51], the best integration interval for calculating the RMS statistic in the monitoring of the grinding process is 1 ms. The RMS statistic is defined by Equation (1).

(1) $$X_{rms}=\sqrt{\frac{1}{N}*\overset{N}{\underset{i=1}{\sum }}x^{2}\left(i\right)}$$

where *x* is the raw signal and *N* is the number of discrete samples (*i*) considered in the calculation.

On the other hand, the Counts statistic was defined by Lopes et al. [9] as the number of times that the signal crosses a threshold per unit of time. The Counts statistic, calculated from the AE signal, has often been implemented in scientific works with different purposes, such as the location and measurement of acoustic emission events in snow blocks before their fracture [52] and the on-line monitoring of abrasive water cutting processes [53].

With regard to the signal analysis, the frequency domain is generally used in identifying events that are difficult to observe in the time domain. The most commonly used frequency domain techniques are the fast Fourier transform (FFT) [54], the short-time Fourier transform (STFT) [55] and the wavelet transform (WT) [56]. The FFT is an algorithm developed to improve the computational efficiency of the discrete Fourier transform (DFT), being a popular method used in spectral analysis and digital signal processing. Ahirrao et al. [57] used the FFT in the analysis of the vibration and dynamics of engines. Kang et al. [58] used the FFT in the tool condition analysis and monitoring of the micro-lens fabrication process.

The statistics presented in this section for the acoustic emission signals can also be used for signals of different natures acquired by other sensors, such as accelerometer and PZT. This work proposes the application of the RMS and Counts statistics in the signals sampled by a set of piezoelectric diaphragms in the emitter-receiver configuration for the monitoring of the material removal in the grinding process. It is worth mentioning that the implementation of the piezoelectric diaphragm in this configuration and for this purpose is unprecedented in the literature.

## 4. Bases of the Chirp-through-Transmission Ultrasound Technique

Nowadays, the inspection of structures by means of ultrasound techniques is widely used. A continuous effort of researchers and industries has been made in order to improve and increase the applicability of non-destructive evaluations (NDE). Thus, a new inspection technique based on ultrasound waves and low-cost piezoelectric transducers is presented in this paper. Section 4.1 presents some basic concepts of ultrasound waves, while Section 4.2 presents some common classifications of these waves. Finally, Section 4.3 presents the method proposed in this paper.

### 4.1. Ultrasound Waves and Their Parameters

Ultrasound signals are used to predict the behavior of materials (detecting internal defects) and to characterize them in a variety of structures. The interest of the scientific community for ultrasound techniques has increased in recent years due to its wide range of applications [41]. Immersion testing and ultrasound contact methods are very popular NDE techniques for characterizing material conditions [59]. However, this type of technique requires the removal of the structure, causing significant interruptions in the operation of the machines [60].

Ultrasound waves are composed of frequencies greater than the human’s hearing range, which is typically 20 kHz [41,42,61]. The main characteristics of the ultrasound waves are length, velocity, pressure, frequency and period. The propagation of the waves in the structure results in two phenomena—velocity alteration and wave attenuation, both caused by the mechanisms of absorption and dispersion [42]. As the ultrasound waves propagate through the material, their energy is diminished and reflections are generated on the surface. These reflections are used to determine the presence and location of discontinuities and defects [61].

The wave velocity is the product between the frequency and the wavelength, so high-frequency waves have shorter wavelengths while low-frequency waves have longer wavelengths [42]. The velocity of the ultrasound waves (v) is determined by the density (ρ) and elasticity (E) of the propagation medium according to the Newton-Laplace equation.

(2) $$v=\sqrt{\frac{E}{\rho }}$$

Equation (2) implies that the velocity of an ultrasound wave in a solid material is greater than in a liquid [42].

There are other ultrasound parameters that correlate the physical-chemical properties of the materials, such as the attenuation coefficient and the acoustic impedance. The attenuation is a result of the energy reduction caused by the compression and decompression of the ultrasonic waves due to the absorptions and dispersions of the propagation medium [62]. Absorption is mainly associated with homogeneous materials while dispersion is related to heterogeneous materials. In addition, attenuation is affected by viscosity, compressibility, material contours and absorption and dispersion effects [42]. It is worth mentioning that the attenuation coefficient of a given material is highly dependent on the way in which the material was manufactured, being useful for product quality control.

Acoustic impedance is the product between the density and the velocity of the wave that passes through the contours of different materials. Materials with different densities have different acoustic impedances, resulting in reflections on the contours between two materials with different acoustic impedances. The attenuation and the acoustic impedance are expressed by Equations (3) and (4), respectively.

(3) $$A=A_{o}e^{-ax}$$

(4) $$R=\frac{A_{T}}{A_{t}}=\frac{z_{1}-z_{2}}{z_{1}+z_{2}}$$

where $A_{o}$ is the initial amplitude of the wave, x is the distance traveled; R is the ratio of the amplitude of the reflected wave ($A_{T}$) to the incident wave ($A_{t}$); $z_{1}$ and $z_{2}$ are the acoustic impedances of two materials.

### 4.2. Classification of Ultrasound Waves

Elastic waves (ultrasonic, sonic and subsonic) can be classified into two groups—body waves, also known as bulk waves and surface waves, also known as guided waves. Body waves propagate through the interior of the material (bulk material), while surface waves propagate along the material’s surface [41].

Bulk waves are classified into two types—longitudinal waves and shear waves. According to Coramik et al. [61], longitudinal waves are also known as compression or extension waves, since the material undergoes compression and extension when the wave propagates through it. In shear waves, the motion of the particles is perpendicular to the direction of the wave propagation. Shear (transverse) waves, when compared to longitudinal waves, have shorter wavelengths and slower dispersion. In terms of elastic constants, the wave velocities of these two types of waves (longitudinal and shear) are given by Equations (5) and (6), respectively [41].

(5) $$c_{p}=\sqrt{\frac{\lambda +2\mu }{\rho }}=\sqrt{\frac{E\left(1-v\right)}{\rho \left(1+v\right)\left(1-2v\right)}}$$

(6) $$c_{s}=\sqrt{\frac{\mu }{\rho }}=\sqrt{\frac{E}{2\rho \left(1+v\right)}}$$

where $c_{p}$ and $c_{s}$ are the longitudinal and shear velocities, respectively. ρ is the density, λ is the first Lame constant, μ is the shear modulus or second Lame constant, E is Young’s modulus and v is Poisson’s ratio. It should be noted that $c_{p}$ is always greater than $c_{s}$, since longitudinal waves propagate faster than shear waves, longitudinal waves are called primary waves or P-waves and shear waves are called secondary waves or S-waves.

### 4.3. The Chirp-Through-Transmission Ultrasound Technique

As already described, the ultrasound wave transmission-reception techniques with transducers not directly coupled to the material are pulse-echo, pitch-catch and through-transmission [41]. In the pulse-echo technique, a single transducer receives and transmits the ultrasound waves. In the pitch-catch and through-transmission techniques, the transmitter and receiver are different transducers [62]. The method proposed in the present study is an alternative to the through-transmission technique, as it also uses two piezoelectric transducers at the ends of the material under study. However, a chirp signal is injected into the emitter transducer, which results in an acoustic wave that propagates through the material until it is collected by the receiver transducer. The signal is collected by a data acquisition system and processed using digital signal processing techniques in order to diagnose the condition of the structure.

**Figure 2:**
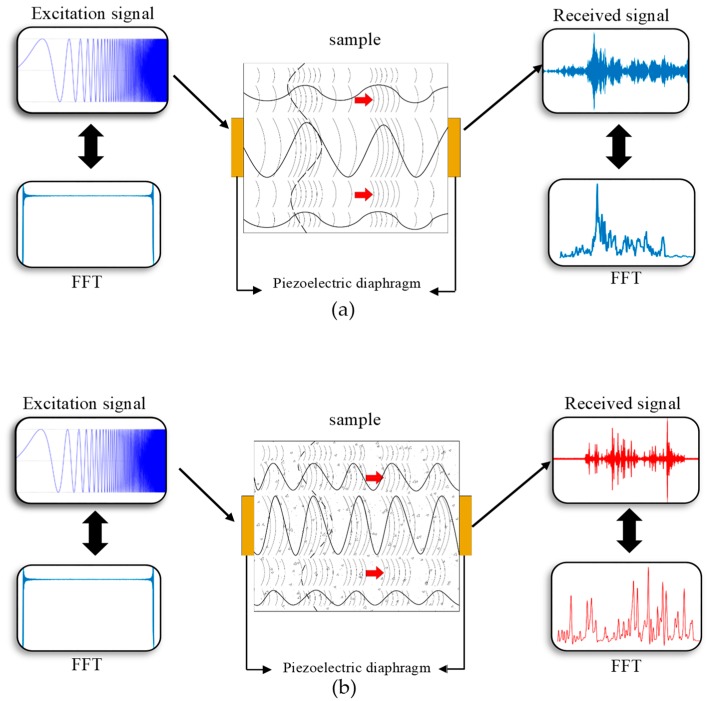
Chirp-Through-Transmission Technique: (**a**) health structure; (**b**) damaged structure.

The proposed method is shown in Figure 2, which shows two piezoelectric diaphragms attached at the ends of the material under study, where the right piezoelectric diaphragm was configured as emitter and the left diaphragm as ultrasonic wave receiver. The emitter is excited by a chirp signal of certain amplitude and frequency. Due to the piezoelectric effect of this transducer when excited with the chirp signal, ultrasonic bulk waves are generated and transmitted through the material under study, as illustrated in Figure 2a. The waves that propagate through the material suffer various effects, such as frequency variations, dispersion, reflection, refraction, compression, rarefaction and energy loss. These effects modify the emitted signal, creating a signature of the material under study. The frequency of the waves changes longitudinally (continuous line) and transverse (dashed line) as they propagate through the structure. Figure 2a shows the application of the technique in a healthy structure (without damage), together with the received signal and its respective frequency spectrum.

In the case of a damaged structure, as shown in Figure 2b, where the points and triangles represent, for example, cracks and material removal, the changes in the waves are different from those observed in the healthy structure and therefore its condition can be diagnosed.

Thus, the signal collected from the damaged material will differ from the signal collected from the healthy material, since changes in structure affect the propagation of ultrasound waves. After the signal acquisition, it is possible to identify the structural change of the material by comparing the signals of the healthy and damaged material. However, this identification involves digital signal processing techniques, such as spectral analysis, frequency band selection, RMS and Counts statistics, among others. Figure 2b shows the application of the technique in a damaged structure, together with the received signal and its respective frequency spectrum, both with different characteristics with respect to the healthy structure.

Thus, the proposed method is based on the changes that ultrasound waves undergo when propagating through the material under study, with the difference that this method does not use the traditional parameters commonly used in ultrasound techniques to diagnose the condition of the structure. Instead, the proposed method employs the processing of the receiver transducer signals resulting from the ultrasound waves generated by the emitting transducer. It is worth mentioning that the prevalent waves in this method are bulk waves, which are expected to allow the monitoring of defects such as microstructure alteration or material removal.

The main features of the proposed method are: (1) The use of a chirp signal at the emitter transducer input instead of pulses; (2) Do not depend on the reflection of the emitted wave; (3) Not limited to specific wavelengths to identify damage; (4) Does not require the use of traditional parameters of ultrasound techniques for damage detection, such as wavelength, propagation speed and time of flight; (5) It is possible to identify the frequency bands in the received wave that best characterize the structural damage; (6) Damage detection can be performed using traditional statistics such as RMS and Counts, enabling the study of many other statistics, indexes and parameters used in process monitoring and SHM; (7) Employs low-cost piezoelectric transducers; (8) It consists of a simple experimental setup for both transducer fastening and data acquisition and signal processing system.

It is worth mentioning that in the proposed method, the changes that the ultrasound waves suffer when propagating through the structure are analyzed through digital signal processing, being necessary the comparison of two signatures, the first of the healthy structure, called baseline and the second one of the altered or damaged structure. Thus, the present method can also be classified as a structural health monitoring method.

According to Giurgiutiu et al. [63,64], the electromechanical impedance (EMI) technique considers the direct and inverse electromechanical properties of piezoelectric materials. In addition, according to Baptista et al. [65], the basic concept of this technique is to monitor structural integrity by exciting a piezoelectric transducer attached to the structure at an appropriate frequency range. Due to the piezoelectric effect, there is a relationship between the mechanical impedance of the structure (related to its integrity) and the electrical impedance of the piezoelectric transducer, obtained by the relationship between the excitation and detection signals. The proposed method, when compared to the EMI technique, does not require the measurement of the structure impedance to detect damage but the variations of the received ultrasonic waves. In addition, common digital signal processing statistics are used to detect differences between the baseline and damage signatures, the technique is not limited to the damage indices commonly used in the EMI technique [66]. Thus, the proposed technique can be expanded to the application of new indices. Finally, traditional EMI techniques use a single sensor to excite and detect the impedance variations of the structure, while in the presented method two transducers are used, analyzing only the received signal.

## 5. Setup, Grinding Tests and Workpiece Assessment

In order to validate the proposed ultrasound method, this section presents the experimental setup for the grinding tests, the workpiece evaluation and the digital signal processing.

### 5.1. Experimental Setup

The grinding tests were performed in a surface grinding machine from Sulmecânica manufacturer, model RAPH 1055 (Porto Alegre, Brazil), equipped with an aluminum oxide grinding wheel, model 38A150LVH, from NORTON company (Worcester, MA, USA). AISI 4340 steel workpieces with 150 mm length × 7 mm width × 43 mm height were machined under three different depth of cuts (*a*). Each test consisted of three grinding passes across the workpiece surface at a given grinding condition. The depth of cut, grinding passes, cutting speed (*v_s_*) and workpiece speed (*v_w_*) are shown in Table 1. The cutting fluid used in the tests was an oil-water emulsion with a concentration of 4%. Prior to test 1, the grinding wheel was dressed in order to restore its topography and cutting potential. In addition, some grinding passes were necessary to standardize the contact between the grinding wheel and the entire length of the workpiece surface. It is worth mentioning that the workpiece surface standardization operation is commonly used in the grinding process since the workpiece was undergone other manufacturing processes prior to the grinding process. These processes, such as milling and turning, hardly achieve the surface finish required by the specifications, thus making the surface of the workpiece uneven to the full contact of the grinding wheel, justifying the need of the surface standardization operation.

**Figure 3:**
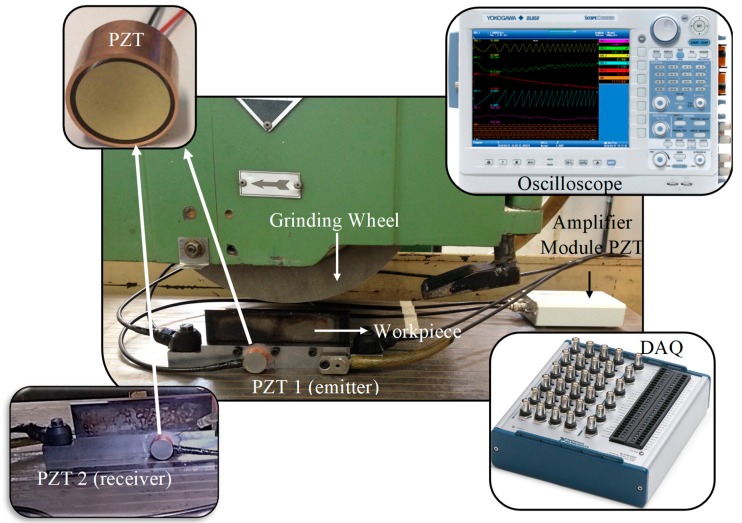
Experimental Setup.

Regarding the monitoring of the process, two piezoelectric diaphragms (buzzers) were attached to the workpiece holder on opposite sides. Prior to the tests, the sensors and data acquisition system were properly calibrated for good signal sensitivity and saturation. The piezoelectric diaphragms used in the tests consisted of a disc-shaped lead zirconate titanate (PZT) ceramic (active element) with a diameter of 10 mm concentrically adhered to a brass disc with a diameter of 20 mm. In addition, the transducers were placed in a metallic case that allowed for a magnetic attachment to the workpiece holder. The experimental setup is shown in Figure 3. Some sensors besides the piezoelectric diaphragm (PZT) can be seen in Figure 3, however, such sensors were not considered in this work. It can also be observed in Figure 3 the metallic case and its connections of the emitter PZT 1 and receiver PZT 2; the latter was attached on the opposite side of the workpiece holder. In order to prevent displacement, a silicone layer was applied around both sensors. Regarding the position of the sensors, both transducers were fixed at a distance of 95 mm from the end of the holder. The transducers have a capacitance of 10 nF and a resonance impedance of 350 Ω, according to the manufacturer’s data. Finally, the interface between the surfaces of the transducer case and the workpiece holder was filled with oil. It is worth mentioning that a thin layer of silicon was placed around the piezoelectric transducer in order to protect it from the cutting fluid and prevent displacements during the grinding process.

The raw signal acquisition was carried out by an oscilloscope, model DL850, from Yokogawa company (Tokyo, Japan), at a sample rate of 5 MS/s. An amplifier model AD8606 (Analog Devices, Norwood, MA, USA), with simple input and gain of 50, was used to amplify the signals from the piezoelectric diaphragm set as receiver. A data acquisition system (DAQ), USB-6361 model, from National Instruments (Austin, TX, USA), was employed to generate the excitation signal of the PZT diaphragm set as emitter. The excitation signal consisted of a chirp interval of 500 ms with a frequency variation from 1 to 250 kHz. In addition, five equal packages containing the chirp signal were sent to the emitter PZT. Thus, the excitation signal was composed of five equal chirp packages sent at 250 ms intervals. The PZT excitation by various chirp signals allows the verification of the repeatability of the results of the digital signal processing. Finally, the emitter PZT was connected to the DAQ which generated the excitation signals (chirp) and the receiver PZT was connected to the signal conditioner (amplifier module). Then, the signal conditioner and the DAQ were connected to the oscilloscope in order to collect the emission and reception raw signals.

### 5.2. Workpiece Assessment

The workpieces assessment was carried out prior to the grinding tests, that is, the workpiece mass was measured before the material removal caused by the grinding process. Thus, the data obtained shows the workpiece characteristics without material removal. After the grinding process (normalization and material removal), the workpieces were reweighed. Thus, it was possible to detect the mass variation of the workpiece machined by the grinding process. A precision scale with a scale of 500 g was used for this purpose. The workpieces were placed in the same position on the precision scale in order to avoid any changes that do not belong to the material removal caused by the grinding process.

### 5.3. Signal Processing and Selection of Frequency Bands

The data sets from the grinding tests were digitally processed in MATLAB software. Two distinct signals were acquired in the tests, the first signal refers to the set of chirp waves of the emitter PZT, while the second signal refers to the chirp waves collected by the receiver PZT. The emission was composed of five chirp signals sent by the emitter PZT and the reception was composed by fives signals acquired by the receiver PZT. The signals collected by the receiver, for each grinding pass and depth of cut, were divided into data vectors or packages, resulting in five packages.

The spectral analysis of the packages corresponding to the receiver signals was performed in order to find the frequency band that best characterize the workpiece conditions (without material removal and with material removal). The Fast Fourier Transform (FFT) was used to analyze the spectral content of the receiver PZT signals. The FFT magnitude was computed for each received vector and then the mean values were obtained. The criterion used for the selection of frequency bands was presented by Ribeiro et al. [16], in which the best frequency bands are those with the greatest differences in magnitudes and minimum overlap between the observed conditions.

After the selection of frequency bands, Butterworth digital filters were applied to the received packages and new vectors were obtained. Then, the RMS and Counts values were calculated for both vectors (raw unfiltered and filtered received signals). Intervals of 4096-point, corresponding to 1 ms, as reported by Webster et al. [51], were used to compute the RMS and Counts values.

**Figure 4:**
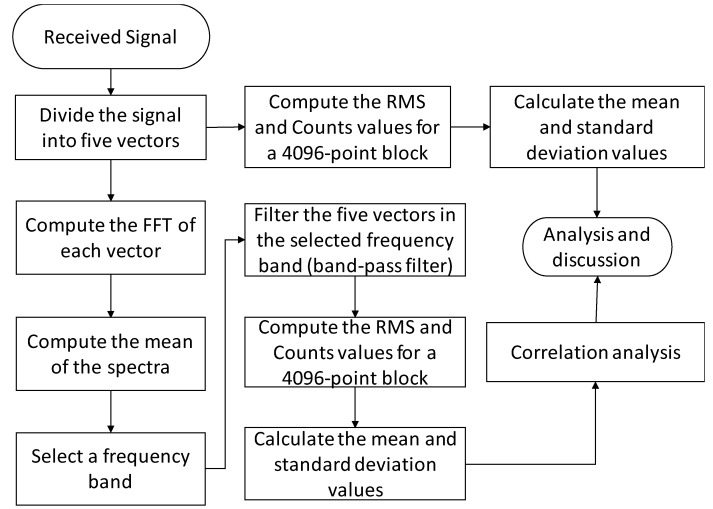
Flowchart of the digital signal processing scheme.

Finally, the standard deviation and mean values for each received package and workpiece condition (without material removal, first grinding pass, second grinding pass and third grinding pass) were computed. It is worth mentioning that the threshold used in the Counts statistic was selected based on the noise level of the receiver signals. The flowchart presented in Figure 4 shows the digital signal processing scheme. In order to correlate the mean values of the statistics (RMS and Counts) with the volume of material removed, a linear regression was calculated. Thus, through the coefficient of determination (*R*) it was possible to infer the degree of correlation between the values obtained with the proposed technique and the volume of material removed. The values were normalized before the computation of the linear regression in order to eliminate the amplitude differences between the two statistics. The application of linear regression as a correlation metric can be found in several scientific works, as in de Oliveira Conceição Jr. et al. [67] and Viera et al. [21].

## 6. Results and Discussion

In this section, the results and discussion of the workpiece assessment and the digital signal processing will be presented, which focus on the correlation of the material removal of the grinding process with the statistics herein applied to the ultrasound signals.

### 6.1. Workpiece Assessment

The variation of masses and weights of the workpieces, measured by a precision scale, before and after the grinding passes, are shown in Table 2. Before the grinding tests, each workpiece underwent the standardization operation, which consists of grinding passes across the workpiece surface (with a depth of cut of 5 µm); the operation was repeated until uniform contact was achieved between the workpiece surface and the grinding wheel surface. After the standardization operation and the grinding passes, the workpiece mass decreases due to the material removal during the grinding process. It is worth mentioning that the mass decrease is due to the standardization and grinding passes. Thus, workpiece 2 had a smaller decrease in mass than workpiece 1 and 3 because the standardization operation was performed with fewer grinding passes than for the other workpieces (1 and 3).

**Figure 5:**
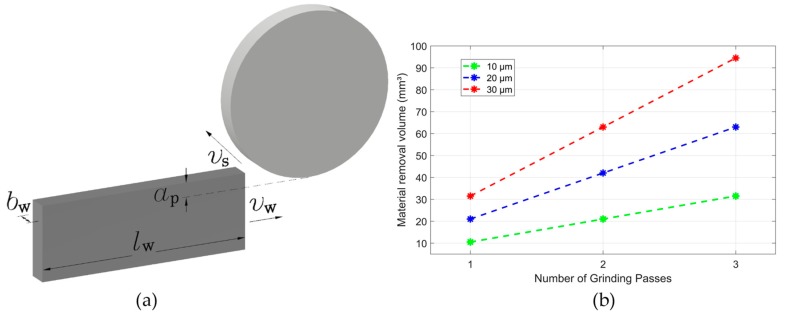
(**a**) Schematic for grinding process with cutting parameters; (**b**) material removal volume as a function of the grinding pass.

The volume of material removed is shown in Figure 5. There is an increase in the volume of material removed according to the number of grinding passes and the selected depth of cut, as expected. The volume of material removed is obtained by Equation (7).

(7) $$Q_{w}\left(p\right)=p*a_{p}*l_{w}*b_{w}$$

where $Q_{w}$ is the volume of material removed in mm^3^, p is the number of grinding passes, $a_{p}$ is the depth of cut in mm, $l_{w}$ is the workpiece length in mm and $b_{w}$ is the workpiece width in mm, as presented in Figure 5a. The volume of material removed as a function of the number of passes is shown in Figure 5b.

### 6.2. Signal Processing and Selection of Frequency Bands

**Figure 6:**
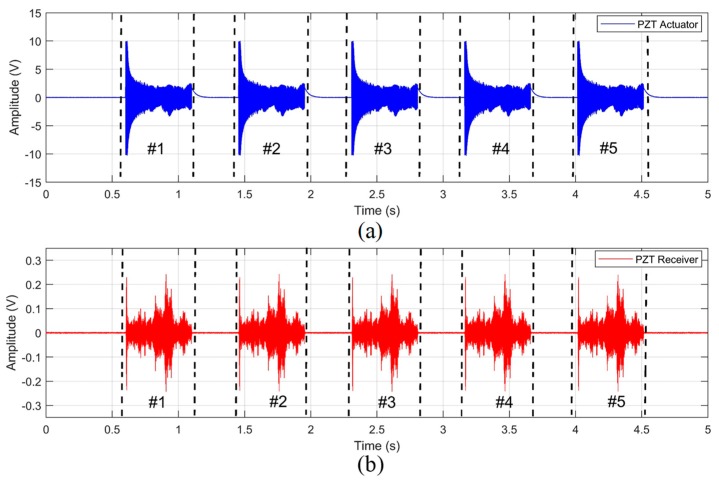
(**a**) Emitted signals packages and (**b**) received signals packages.

The emitted and received signals used to evaluate the workpieces are shown in Figure 6. It is observed in Figure 6a,b that the emitted and received signals have five packages (#1, #2, #3, #4, #5), respectively. The amplitude of each emitted package decreases as the frequencies increases; this behavior can be explained by the arrangement of the impedance between the DAQ (chirp signal emitter) and the emitter PZT. The PZT capacitive elements caused a voltage decrease at higher frequencies, as observed in Campeiro et al. [68]. Regarding the received packages in Figure 5b, it is observed that some frequencies propagated with greater effectiveness; this behavior is justified by the material characteristics that play a very important role in the propagation of waves. It is worth mentioning that the emission packages started with amplitudes of 10 V and finished with amplitudes of 3 V, whereas the received packages started with amplitudes of 200 mV, having in certain times amplitudes of 50 mV. This significant attenuation is due to the energy loss through the PZTs’ intrinsic characteristics, the workpiece holder medium, interfaces (PZTs/holder; workpiece/holder), coupling medium, workpiece medium, amplifier and cables. However, despite the high energy loss of the ultrasound signal for the setup used in this work, the results will later show that the received signal preserved the process characteristics under study. The procedure for the emitted and received packages shown in Figure 6 was repeated for each grinding pass at each selected depth of cut.

**Figure 7:**
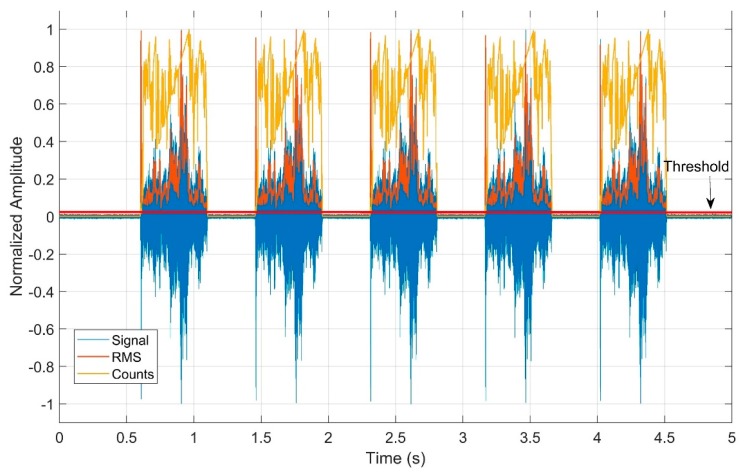
Statistics used to analyze the received packages—root mean square (RMS) and Counts.

The statistics used in the analysis of the received packages are shown in Figure 7. It is worth mentioning that, in order to show the statistics together, it was necessary to normalize the values. However, the analyses, such as the mean and standard deviations, were performed with the raw unfiltered signals without the normalization procedure. The analyses were performed for each package considering the mean values of these statistics (RMS and Counts). Subsequently, a total mean value and standard deviation, with respect to all packages, was calculated.

**Figure 8:**
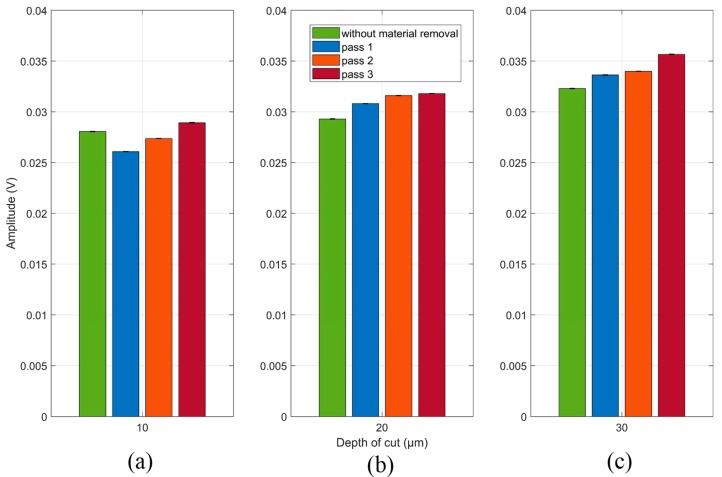
RMS mean and standard deviation values of the raw unfiltered signals at (**a**) 10 µm, (**b**) 20 µm and (**c**) 30 µm.

The RMS mean and standard deviation values (unfiltered signals) for each grinding pass at each depth of cut are shown in Figure 8. In Figure 8a it can be seen that the result of the RMS mean value without material removal showed a greater amplitude compared to the results of the grinding passes 1 and 2. This non-linear behavior hinders the implementation of this system in the diagnosis of material removal during the grinding process. In relation to Figure 8b,c, a growth tendency is observed as the grinding passes occur; however, the difference of the RMS mean values between the 2nd and 3rd grinding pass for the 20 µm depth of cut is very small, making it difficult to identify the grinding pass and thus, being unattractive for practical implementation. In the same way, this analysis can be applied to the RMS mean values of the 1st and 2nd grinding passes for the 30 µm depth of cut in which there is a small difference, making it difficult to identify this grinding passes. It is worth mentioning that the standard deviations, compared to the RMS mean values, were very small, around 1%, for all the observed conditions. This behavior characterizes the consistency and repeatability of the emission and reception signals used in the proposed technique.

**Figure 9:**
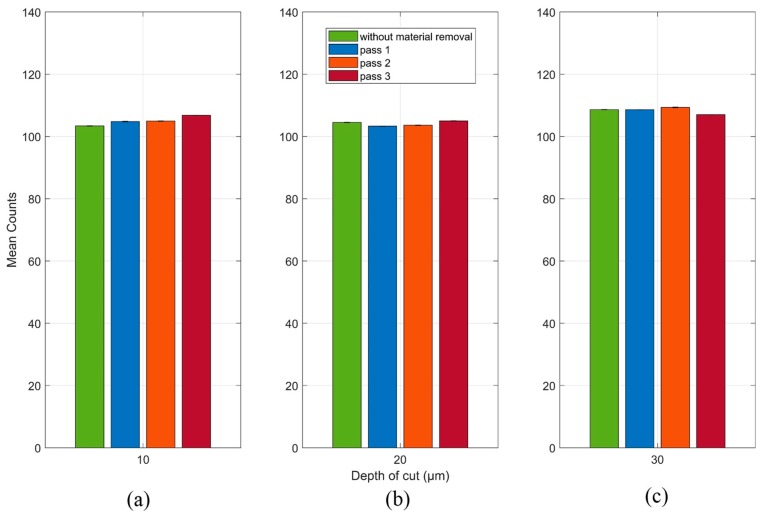
Counts mean and standard deviation values of the raw unfiltered signals at (**a**) 10 µm, (**b**) 20 µm and (**c**) 30 µm.

The Counts mean values and the standard deviations (unfiltered signals) for each grinding pass at each depth of cut are shown in Figure 9. As in the behavior shown by the RMS results, the application of the Counts statistic was not effective in identifying a linear growth pattern between the grinding passes. The depth of cuts of 20 µm (Figure 9b) and 30 µm (Figure 9c) showed a non-linear behavior. In Figure 9b it is observed that the Counts mean value of the undamaged workpiece is higher than the Counts mean values for the 1st and 2nd grinding pass. Regarding Figure 9c, the Counts mean value of the workpiece without damage had a very close result in relation to the 1st and 2nd grinding pass, however, it was higher than the Counts mean value of the 3rd grinding pass. Finally, regarding Figure 9a with a 10 µm depth of cut, an increasing pattern was observed, however, the difference between the 1st and 2nd grinding pass was small, similar to the behavior shown by the RMS mean values at the depth of cut of 20 µm (Figure 8b) and 30 µm (Figure 8c). The standard deviations, when compared to the mean values, were very small, around 1%, similar to the values found in the RMS results, shown in Figure 8. Again, the behavior of the presented results emphasizes the consistency and repeatability of the technique used in this work. Both statistics (RMS and Counts) did not show a regular tendency in the diagnosis of material removal, thus a spectral analysis of the received signals for each grinding pass was performed in order to find the frequency bands that are more strongly related to the process conditions.

**Figure 10:**
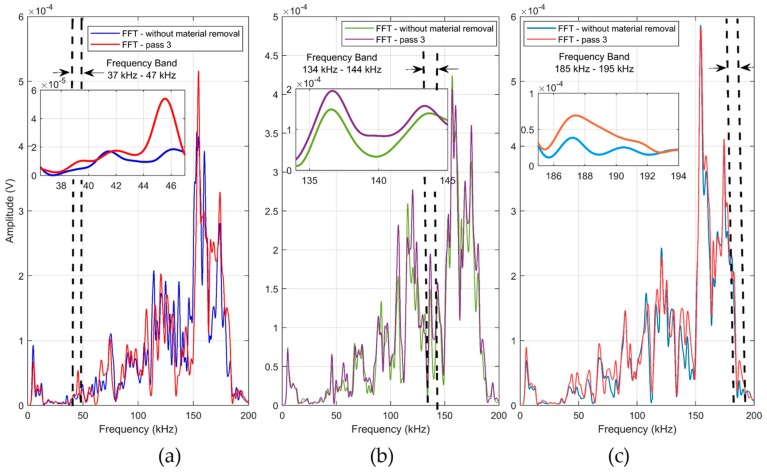
Spectrum of two workpiece conditions at (**a**) 10 µm, (**b**) 20 µm and (**c**) 30 µm.

The mean spectrums for two workpiece conditions, without material removal and after the 3rd grinding pass, at each depth of cut, are shown in Figure 10. It can be seen in Figure 10 that the spectrums, in general, showed similar behavior, with higher spectral activity between 150 and 190 kHz.

**Figure 11:**
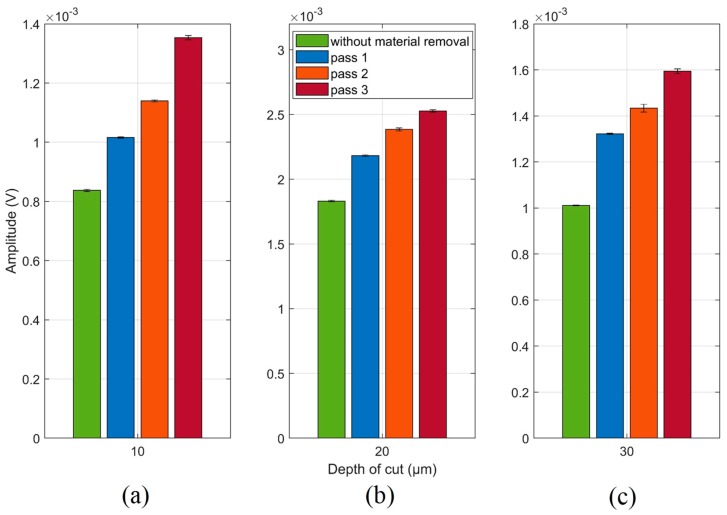
RMS mean and standard deviation values of the raw filtered signals at (**a**) 10 µm; (**b**) 20 µm and (**c**) 30 µm.

Furthermore, it is observed that the spectrum of the 3rd grinding pass at the three depth of cuts, 10 µm (Figure 10a), 20 µm (Figure 10b) and 30 µm (Figure 10c), presented the greatest amplitudes along most part of the spectrum when compared with the workpiece without material removal. By means of the frequency band selection criterion, presented in Section 5.2, the 37 to 46 kHz frequency band was selected and subsequently filtered into the received packages of the 10 µm depth of cut, as shown in the magnification of Figure 10a. For the 20 µm depth of cut, a frequency band from 135 to 144 kHz was selected, as shown in Figure 10b. Regarding the 30 µm depth of cut, a frequency band of 186 to 192 kHz was selected. The frequency bands magnifications of both depth of cuts (20 and 30 µm) are shown in Figure 10b,c, respectively. The RMS mean values and standard deviations of the filtered signals for each grinding pass at each depth of cut are shown in Figure 11. In contrast to the results observed in Figure 8, the filtered RMS values showed a uniform increase trend. An increase in the RMS values was identified according to the grinding passes. The increase in the RMS values was due to the behavior of the amplitude of the signals in the selected frequency bands. After the application of digital filters in the selected frequency bands and subsequent computation of the RMS statistic, an increasing trend was observed at all depths of cut (Figure 11a–c). This behavior can be explained by the signal amplitudes in the selected bands, that is, the changes in the workpiece (material removal) presented higher amplitude levels [26]. It is worth mentioning that the sample changed after each grinding pass and, as a consequence, the propagated waves also showed changes that are evidenced in the selected frequency bands. Thus, events produced during the grinding process that indicated changes in the workpiece structure, such as material loss, burning, high roughness and cracks, were evidenced in the selected frequency bands, allowing better diagnosis of the process. Finally, it is worth mentioning that the standard deviation values remained small, again showing the consistency and repeatability of the method. Thus, the appropriate selection of frequency bands is very important for the application of this technique.

**Figure 12:**
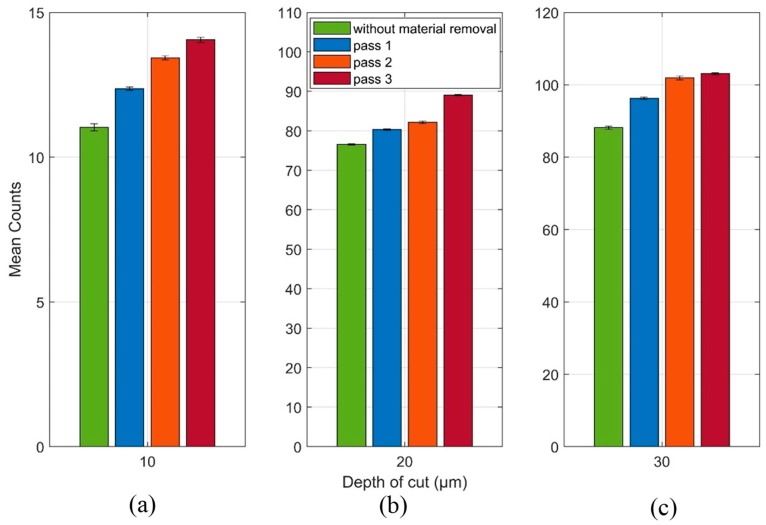
Counts and standard deviation values of the raw filtered signals at (**a**) 10 µm; (**b**) 20 µm and (**c**) 30 µm.

The Counts and standard deviation values of the filtered signals for each grinding pass at each depth of cut are shown in Figure 12. As with the filtered RMS values shown in Figure 11, the mean Counts values for the same signals showed an increasing trend in all the conditions observed. However, it is worth mentioning that the depth of cut of 10 µm shown the best result for this statistic, whereas in the depths of cut of 20 and 30 µm the Counts presented a small improvement when compared to the optimal result obtained in the RMS application of Figure 11. Thus, the Counts and RMS statistics can be used together, with priority for the RMS due to the optimal results; the Counts statistic can be used with the purpose of validating the RMS results and strengthening the damage diagnosis system in the grinding process.

**Figure 13:**
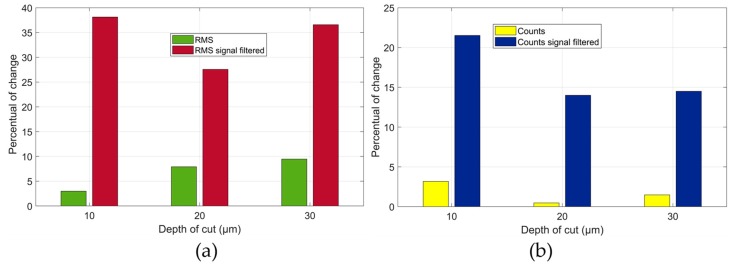
Percentage variation between the unfiltered and filtered signals of the workpiece without removal material and after the 3rd grinding pass—(**a**) RMS and (**b**) Counts values.

The percentages of difference between the mean RMS and Counts values of the unfiltered and filtered packages for two workpiece conditions, without material removal and after the 3rd grinding pass, at each depth of cut are shown in Figure 13. In Figure 13a it can be observed that the highest percentage variation is around 38% for the filtered signal and depth of cut of 10 µm. Similarly, the percentage of difference for the Counts mean values, shown in Figure 13b, presented the greatest difference at the same depth of cut, around 22%. Finally, the differences in the observed conditions are higher in the results of the mean RMS, denoting that the RMS statistic is more adequate for the application of this technique due to the better results.

As demonstrated by Webster et al. [51], the 1 ms interval is best suited for calculating the RMS value in the monitoring of grinding processes. The same interval, corresponding to 4096 points, was applied in the Counts statistic. Thus, it is possible that this interval is not the most appropriate for this statistic, which explains the better result of the RMS statistic when compared to Counts. In addition, the choice of the most appropriate frequency band is crucial for the performance of the statistics. Therefore, other intervals and frequency bands can be studied in order to optimize the Counts statistics.

**Figure 14:**
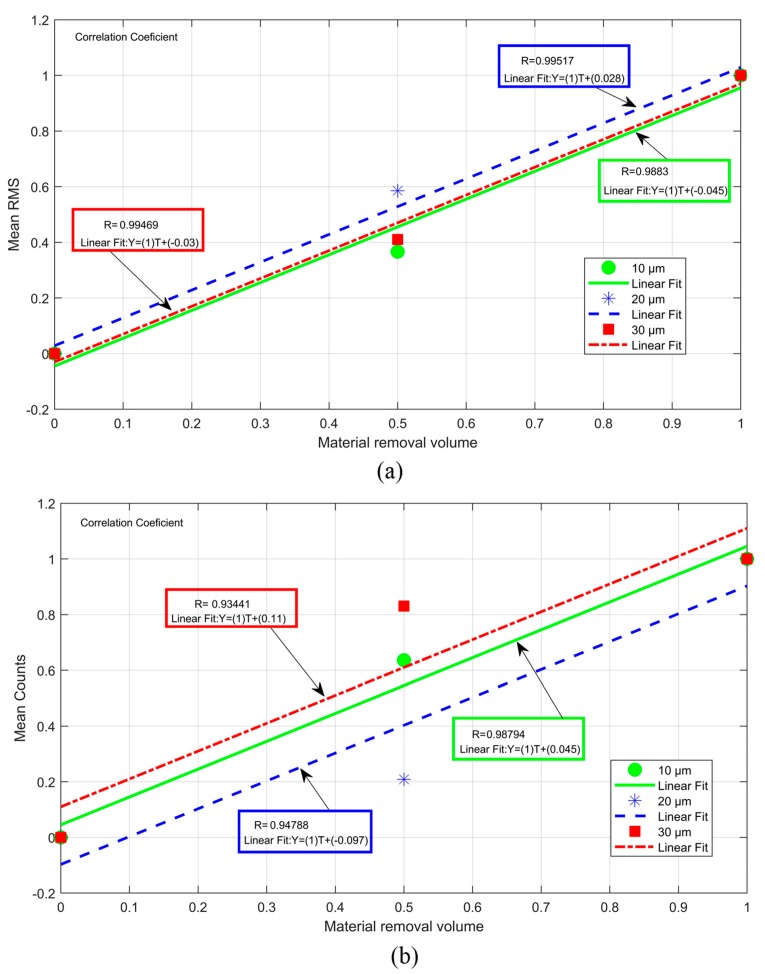
Correlation between the statistics and the volume of material removed (**a**) RMS and (**b**) Counts.

The correlations between the mean values of the statistics (RMS and Counts) and the volume of material removed are shown in Figure 14. The correlation analysis is performed by means of the coefficient of determination (*R*), where *R* = 1 represents a linear fit of 100% and *R* = 0 represents the complete lack of correlation between the values [21]. It can be observed that for the RMS statistic (Figure 14a), the *R* values were close to 1. Regarding the Counts statistic (Figure 14b), the coefficient of determination showed a high degree of correlation; however, the coefficient was lower than the coefficient found for the RMS statistic. At the 10 µm depth of cut, the RMS statistic presented an *R* of 0.9883 and the Counts statistic an *R* of 0.98794. Thus, the RMS was slightly better than the Counts in the estimation of the volume of material removed. At the depth of cuts of 20 and 30 µm, a higher sensitivity of the RMS statistic was observed when compared to the Counts statistic.

At 20 µm, the RMS had an *R* = 0.99517 while Counts had an *R* = 0.94788. Finally, at 30 µm the largest difference was observed, where the RMS presented an *R* = 0.99469 and Counts presented an *R* = 0.93441. Thus, in the application of this technique, the RMS statistic has a better performance than the Counts statistic, however, the high degree of correlation shows that both statistics can be used to indirectly determine the volume of material removed.

In a system where both statistics can be applied, it is possible to obtain results that confirm the material removal diagnoses, since both statistics showed the same increasing trend. It is worth mentioning that, in both statistics, the filtered signals produced a much greater percentage of change when compared to the unfiltered signals. Thus, the selection of frequency bands that best characterize the events occurred during the grinding process is very important for the application of the technique proposed in this work.

## 7. Conclusions

The interest of the scientific community in ultrasound techniques has increased in recent years due to its wide range of applications. Thus, a new method of ultrasound wave monitoring is proposed in this study as an alternative to traditional ultrasound methods. The main advantage of this method is the use of low-cost transducers for the emission and reception of ultrasound waves in conjunction with traditional signal processing statistics, thus allowing the non-invasive monitoring of structures. Unlike traditional methods of ultrasound analysis, the method presented in this paper does not depend on reflections and wavelengths to identify damage. Moreover, the proposed method does not require the use of the traditional ultrasound parameters (wavelength, propagation velocity and time of flight) to detect changes in the structures. Finally, this method uses traditional statistics for monitoring the material removal volume in grinding, allowing the study of other statistics, indexes and parameters, such as RMSD and CCDM used in SHM.

A study of the frequency bands that best correlate with the workpiece characteristics during the grinding passes was performed. Thus, it can be concluded that the unfiltered RMS and Counts mean values have low sensitivity to the material removal changes and are not attractive for practical purposes. On the other hand, the RMS and Counts mean values of the filtered signals in the selected frequency bands showed excellent results, obtaining an increasing tendency according to the number of grinding passes that occurred in the tests. It is worth mentioning that the filtered RMS values presented the best results when compared with the Counts values in the same condition. The highest percentage changes between the workpiece without material removal and after the 3rd grinding pass for each depth of cut was observed for the mean RMS values. The frequency band selection and the interval period were determinant for the sensitivity of the statistics. In addition, the correlation analysis between the statistics (RMS and Counts) and the volume of material removed reinforced the results obtained, proving the viability of applying the chirp-through-transmission technique in the monitoring of material removal, as all the coefficients of determination were higher than 90%.

Nevertheless, the results presented in this work are preliminary and new studies must be conducted for improvements of the proposed technique with application in grinding, SHM as well as in other machining processes.

## Figures and Tables

**Table 1:** Grinding test parameters.

Workpiece	Depth of Cut *a* (μm)	Passes	Cutting Speed *v_s_* (m/s)	Workpiece Speed *v_w_* (m/s)
1	10	3	29	0.08
2	20			
3	30			

**Table 2:** Variation of masses and weights of the workpieces.

Workpiece	Condition	Weight (g)	Mass Decrease (%)
1	Without material removal	329.75	0.05
	After pass 3	329.60	
2	Without material removal	327.24	0.02
	After pass 3	327.15	
3	Without material removal	331.24	0.33
	After pass 3	330.15	
